# Rapid Development of Microsatellite Markers with 454 Pyrosequencing in a Vulnerable Fish*,* the Mottled Skate*, Raja pulchra*

**DOI:** 10.3390/ijms13067199

**Published:** 2012-06-12

**Authors:** Jung-Ha Kang, Jung-Youn Park, Hyun-Su Jo

**Affiliations:** 1 Biotechnology Research Division, NFRDI, Busan 619-705, Korea; E-Mails: kjh0124@nfrdi.go.kr (J.-H.K.); jypark@nfrdi.go.kr (J.-Y.P.); 2 Fisheries Resources and Environment Division, West Sea Fisheries Research Institute, NFRDI, Incheon 400-420, Korea

**Keywords:** *Raja pulchra*, pyrosequencing, microsatellite

## Abstract

The mottled skate, *Raja pulchra*, is an economically valuable fish. However, due to a severe population decline, it is listed as a vulnerable species by the International Union for Conservation of Nature. To analyze its genetic structure and diversity, microsatellite markers were developed using 454 pyrosequencing. A total of 17,033 reads containing dinucleotide microsatellite repeat units (mean, 487 base pairs) were identified from 453,549 reads. Among 32 loci containing more than nine repeat units, 20 primer sets (62%) produced strong PCR products, of which 14 were polymorphic. In an analysis of 60 individuals from two *R. pulchra* populations, the number of alleles per locus ranged from 1–10, and the mean allelic richness was 4.7. No linkage disequilibrium was found between any pair of loci, indicating that the markers were independent. The Hardy–Weinberg equilibrium test showed significant deviation in two of the 28 single-loci after sequential Bonferroni’s correction. Using 11 primer sets, cross-species amplification was demonstrated in nine related species from four families within two classes. Among the 11 loci amplified from three other *Rajidae* family species; three loci were polymorphic. A monomorphic locus was amplified in all three *Rajidae* family species and the *Dasyatidae* family. Two *Rajidae* polymorphic loci amplified monomorphic target DNAs in four species belonging to the Carcharhiniformes class, and another was polymorphic in two Carcharhiniformes species.

## 1. Introduction

Many sharks and skates are particularly vulnerable to overfishing because of their large size, slow growth, late maturity and low fecundity. Among the 227 species in 25 genera of the family *Rajidae*, 11 skate species belonging to four genera have been identified in Korea to date. Skates are commercially important in Korea, and the mottled skate, *Raja pulchra*, is the most favored and valuable species because of its superior meat quality [1]. This species is one of the most expensive fishes sold in South Korea, reaching US $10–30 per kilogram. This species is also valued in China and Japan for their dried wings and meat, but is less expensive (US $5) than in South Korea.

*Raja pulchra*, a low-boreal subtropical species abundant in the coastal waters of some areas of Korea and Japan, is usually caught at a depth of about 5–100 m [2–4]. This species has been known to inhabit the East China Sea, the Yellow Sea, the East Sea, the Pacific coast of Japan, the southern Kurils, and a recent survey has shown that they are present off the coast of western Sakhalin [5,6].

Catch data from Korea shows a 90% decline over a ten-year period. The average annual catch from 1991–1993 was 2700 metric tons, whereas the catch in 2011–2003 was 220 metric tons [7], reflecting a serious decline in the biomass collected. In China, there has been an overall reduction in all fish biomass due to overexploitation and heavy trawling within the mottled skate’s range, as well as habitat degradation as a result of such fishing activities. The overall decline in numbers of this species has exceeded 30% over the last 15 years (a period of three generations) [8]. As a result, the International Union for Conservation of Nature (IUCN) has designated it as “vulnerable” and in fact, the decline may be severe enough to warrant a status of “endangered” [8]. However, there are no conservation measures in place for this species. Further assessment and monitoring of catches in fisheries and population trends are required.

Microsatellite (MS) or simple sequence repeat (SSR) markers are versatile molecular tools for determining parentage, inferring genetic structure and gene flow patterns and for assessing the origins of introduced populations [9,10]. Traditionally, the isolation of MS DNA loci has relied on the screening of genomic libraries using repetitive probes and sequencing of positive clones to develop locus-specific primers. While effective in many applications, these cloning and sequencing procedures are tedious, labor-intensive and expensive [11,12].

Next generation sequencing (NGS) technologies have revolutionized many life science fields because of its rapid cost-efficient parallel processing of millions of templates [13]. NGS is capable of generating gigabases of sequence data in a single run, with individual reads long enough (>500 bp) to capture individual microsatellites and the flanking sequence for PCR primer design. Additionally, there is no need for enrichment because even a fraction of a run can provide sufficiently large numbers of random sequence reads to contain many thousands of microsatellites markers. Application of this technique to expressed genes would make it possible to develop panels of MS markers for genes underlying phenotypic variation [14].

Based on these advantages, this new technology is expected to replace conventional microsatellite isolation protocols [15], and there are increasing reports employing NGS MS markers in studies of mammals [16], plants [17,18], insects [19], and amphibians [20]. This new technology also has been applied in the development of microsatellite maker in marine organisms and was proven to be effective [21–24].

In this work, we carried out a global analysis of the repetitive fraction of the *R. pulchra* genome using NGS combined with the bioinformatics approach described above. This work will be useful for future resource management of this economically and ecologically important, but vulnerable, species.

## 2. Results

### 2.1. Pyrosequencing, Repeat Identification and Classification

NGS produced 453,549 reads with an average length of 487 bp; 4,305 contiguous sequences (contigs) were constructed by Newbler (version 2.6; Roche) from these reads. The total number of bases was 2,024,317, and the largest contig was 16,906 bp. In addition, we found 307,931 unassembled singleton sequences for a total read of 150,038,230 bp. The total number of reads after trimming of low quality sequence was 288,854, and the sum of the total reads was 148,321,261 bp in length, which was used in a search for SSRs. The number of reads with dinucleotide repeat were: 4164 AT, 6544 CA, 6239 CT, and 86 GC repeats. The sequence and number of 2625 trinucleotide repeats are shown in Table 1.

### 2.2. Selection and Characterization of the SSR

Thirty two loci containing dinucleotide microsatellites with over nine repeats were selected for further evaluation. Primers were designed for these loci and tested in four individuals. Twenty primer sets (62%) produced strongly amplified PCR products, and 14 showed clear amplification with polymorphic patterns. However, no clear genotype pattern was obtained for five loci, and Rp19-nfrdi was monomorphic in all of the tested individuals. The primer sequences, repeat motifs, annealing temperatures, PCR ranges, number of alleles, and GenBank accession numbers for the 15 new microsatellite loci are summarized in Table 2. The number of alleles per locus varied from one (at Rp19-nfrdi) to ten (at Rp11-nfrdi) in a total of 60 tested individuals.

### 2.3. Genetic Diversity of Raja pulchra Populations

Table 3 summarizes the genetic characterization indices estimated for the two skate ray populations. Allelic richness per locus ranged from 2–9.8 in the two populations. The mean allelic richness was 4.7. The average number of alleles among all populations was 4.8. In total, there were 13 unique alleles, five from the Daecheong-do population and eight from the Heuksan-do population. No linkage disequilibrium was found between any pair of loci (*p* > 0.05), indicating that the markers were independent. The Hardy–Weinberg equilibrium (HWE) test, indicating the deviation from expected heterozygosity, showed significant deviation in two (Rp3-nfrdi and Rp43-nfrdi) of the 28 single-loci in the Heuksando population after sequential Bonferroni’s correction. Null alleles were presumed in four (Rp3, Rp11, Rp18 and Rp43-nfrdi) of 14 loci. No genetic differentiation between DC and HS populations was detected by *F_ST_* (=0.013) using all 14 microsatellite markers.

### 2.4. Cross-Species Amplification

Cross-species amplification of 11 loci was conducted in nine related species belonging to four families within two classes, as shown in Table 4. These are representative species of cartilaginous fish. All of the 11 loci amplified the target DNAs from the three other fish species, *Okamejei boesemani*, *O. kenojei* and *O. acutispina* that belong to the same family, *Rajidae*. Among these loci, Rp11, Rp16 and Rp53 were polymorphic in all three species. The locus Rp16, which was monomorphic in *R. pulchra*, was also monomorphic in all three species. Additionally, the monomorphic locus Rp16 was amplified in two fish species, *Dasyatis akajei* and *Uroplophus aurantiacus*, belonging to the *Dasyatidae* family. In contrast, no amplification of other loci was observed in either or both of these species. The Rp3 and Rp16 loci that were polymorphic in the *Rajidae* family amplified monomorphic target DNAs in four fish species belonging to the class Carcharhiniformes, and the Rp35 locus was polymorphic in two species belonging to this class (Table 4).

## 3. Discussion

Among 11 skate species identified in Korea so far, *R. pulchra* is the most economically important species, and it is consumed raw or as fermented fish. It inhabits coastal areas around the Korean Peninsula and was extremely common about 20 years ago in the Yellow Sea, especially around the Daecheong-do and Heuksan-do Islands of Korea. As in other skates, reproduction of *R. pulchra* is oviparous, and the number of eggs in each spawning is less than 250 [4]. Because there has been a >90% catch decrease over the past ten years, it has been placed on the “vulnerable” species list by the IUCN [8]. Therefore, it is necessary to understand the genetic structure and population diversity for future management and resource restoration.

MS markers have many advantages over other molecular markers for the study of population structure and diversity [25]. However, the cloning and sequencing processes commonly used in MS marker development are time consuming and costly, often with positive clone yields as low as 0.03% [26].

NGS has recently been applied in the development of MS markers in many organisms, greatly reducing the cost, labor and time required [13,15,16,27]. This method has been shown to be more effective when enriched DNA libraries are used for sequencing [28]. Despite the advantages, the development of MS markers using NGS in aquatic organisms has been limited, but has been proven to be effective in recent publications [21–24,29].

In addition to speed and cost-effectiveness, NGS offers high flexibility in primer design because a large number of reads and sequences containing SSRs and loci with the proper repeat units can be selected. In our study, 17,033 (5.5%) from a total of 312,290 contigs contained dinucleotide SSRs, and 33 loci containing a minimum of nine repeat units were used for primer design, which is only 0.2% of the identified SSRs. In the analysis of MS markers reported in plant, only 1% of polymorphic loci identified using NGS were selected by stringent criteria for primer design, compared with 20% of SSRs discovered using a traditional cloning and sequencing process were selected for primer design [30].

To select optimal primers from a large number of candidates, high-quality standards can be applied in loci selection, which can increase the success rate and reduce the time, labor and cost of marker development. For instance, to minimize the risk of null allele amplification, 454 sequence alignments can be used to design primers using loci with a reasonably high depth of coverage at both primer-annealing sites, and that show no evidence of SNPs within the regions [31]. In our study, 20 (62%) primer sets among 32 loci containing a minimum of nine repeat units produced amplified PCR products, and 14 (70%) of them were polymorphic.

After sequential Bonferroni correction for multiple tests, significant deviations from HWE in the direction of heterozygote deficiency were detected at only two (Rp3-nfrdi and Rp43-nfrdi) of the 14 tested loci in the Heuksan-do population. Generally, heterozygote deficiencies increase due to factors such as inbreeding, substructuring of the population sample, or the presence of null alleles. Indeed, our Microchecker analysis revealed the presence of null alleles at four loci, including Rp3-nfrdi and Rp43-nfrdi. The level of genetic diversity (mean *Ho* = 0.57, *He* = 0.61; mean allelic number = 4.7) in this study was slightly higher than that of the common skate, *Dipturus batis* (mean *Ho* = 0.35, *He* = 0.36; mean allelic number = 3.5) [32]. No genetic differentiation between populations by *F_ST_* may suggest that the two populations can be regarded as one population. However, differentiation of genetically related skate population by distance has been observed in northeast Atlantic continental shelf [32]. To reduce the time and cost of MS marker development, pre-existing markers from related species have been applied to the study of many animal, fungus, plant and fish species [33,34]. Moreover, cross-species markers can be used for the identification of invading species and the identification of parental origin in hybrid fish [35]. Although the cross-species transferability of MS markers is unevenly distributed among taxa, over 40% and 25% of polymorphic marker transfers have been observed in fish of different genera within the same family, and of families within same order, respectively [33].

With large numbers of SSR candidates identified by pyrosequencing, the development of cross-species markers has been attempted in diverse organisms [36]. The markers developed in this study were tested for cross-species amplification and possibility of finding universal markers for cartilaginous fish with available fish samples. Among the ten markers polymorphic in *R. pulchra*, four (40%) were polymorphic in other species within same family, which is comparable to previous data [33]. However, none of them showed any polymorphic amplification of targets in specimens belonging to different families within the same species. One interesting finding was that loci Rp16 and Rp35, which do not amplify the target sequences in the family *Dasyatididae*, produced monomorphic or polymorphic PCR products in Carcharhiniformes. Although the numbers of genera and samples for each genus are limited, these results suggest the possibility that cross-species polymorphic markers can be developed in fish species using the pyrosequencing technique with large number of SSR candidates for marker development.

The rapid population decline of *R. pulchra* around the Korean Peninsula, especially in the Yellow Sea, is due to many factors such as overfishing and destruction of their natural habitat. However, there have been no conservation measures in place for this species. Therefore, the molecular markers developed in this study will be useful for future assessment and monitoring of population trends of this species. In addition, this study suggests that pyrosequencing methods will be useful for SSR development in aquatic organisms, and may be preferable to the traditional labor- and time-intensive cloning methods.

## 4. Experimental Section

### 4.1. Sample

A total of 60 wild *R. pulchra* samples were collected around the Korean islands: 30 from the Daecheong-do population and 30 from the Heuksan-do population. Muscle tissue samples were preserved in 100% ethanol at the sampling site and then transported to the laboratory for DNA extraction. Total DNA was isolated from each sample using a MagExtractor MFX-6100 automated DNA extraction system (Toyobo, Osaka, Japan). The extracted genomic DNA was quantified using a Nanodrop ND-1000 spectrophotometer (Thermo Fisher Scientific, Barrington, IL, USA) and stored at −20 °C until genomic DNA pyrosequencing. For the cross-species transferability test, DNA was extracted by the same method from ethanol-fixed tissues of nine related species belonging to four families within two classes that had been stored at the National Fisheries Research and Development Institute, Busan.

### 4.2. DNA Sequencing

DNA sequencing was performed with 454 pyrosequencing on a Genome Sequencer FLX-454 System (GS FLX sequencer). Sample preparation and DNA sequencing was performed according to the manufacturer’s instructions (Roche Diagnostics, Mannheim, Germany). DNA sample was prepared from one individual and sequencing was conducted using a half of a 454 plate at the National Instrumentation Center for Environmental Management (NICEM) of Seoul National University.

### 4.3. De Novo Assembly and SSR Findings

The raw reads from the GS FLX 454 were assembled using the Newbler 2.6 software and the assembled contigs consensus and unassembled singleton sequences were merged to discover SSRs on the genome sequence. The sequence was filtered with high quality score using the Less Useful Chunks Yank (LUCY) 1.20p. A modified “SSR_finder.pl” perl program was then used to detect di- and tri-SSR markers. A pair of primers flanking each SSR was designed using Primer3 software [37]. The primer redundancy was tested by using the BLAST available at NCBI [38] on the basis of ≤0.001 e-values.

### 4.4. PCR and Genotyping

Newly-designed PCR primer pairs were tested to optimize annealing temperatures; a gradient PCR with a 50–60 °C annealing temperature range was performed on a set of samples from eight individuals. PCR amplification was performed in a 10 μL reaction mixture containing 0.25 U *Ex taq* DNA polymerase (TaKaRa Biomedical Inc., Shiga, Japan), 1× PCR buffer, 0.2 mM dNTP mix, 10 pmol each primer (the forward primer of each pair was 5′-end-labeled with 6-FAM, NED, and HEX dyes; PE Applied Biosystems), and 100 ng template DNA, using a PTC 200 DNA Engine (MJ Research, Waltham, MA, USA). PCR conditions were as follows: 11 min at 95 °C, followed by 35 cycles of 1 min at 94 °C, 1 min at the annealing temperature listed in Table 2, and 1 min at 72 °C, with a final extension of 5 min at 72 °C. Microsatellite polymorphisms were screened using an ABI PRISM 3130 XL automated DNA sequencer (Applied Biosystems), and alleles were designated according to PCR product size, relative to a molecular size marker (GENESCAN 400 HD [ROX]; PE Applied Biosystems).

### 4.5. Statistical Analysis

The number of alleles per locus, allele frequency, and heterozygosity were calculated using Arlequin version 3.0 [39]. Tests for population-wide linkage disequilibrium between pairs of loci and deviations from HWE were estimated using GENEPOP version 4.0 [40], and the adjusted *p*-values for both analyses were obtained using a sequential Bonferroni test for multiple comparisons. MICROCHECKER version 2.2.3 [41] was used to test the presence of null alleles. Allelic richness (*A_R_*) as a standardized measure of the number of alleles per locus, independent of the sample size, was calculated using FSTAT version 2.9.3 [42]. A possible geographical pattern in the distribution of genetic variability was analyzed through *F_ST_* estimates and genetic distances between each pair of populations.

## 5. Conclusions

The pyrosequencing method was applied in the development of MS markers for *R. pulchra*, which is listed as a “vulnerable” species by the IUCN. A comparably high primer-to-marker conversion ratio (62%) was achieved by this method. In the cross-species amplification, over 90% of the markers were amplified in the fishes belonging to the same family but the success ratio was much less in the different family. The molecular markers developed in this study can be used for future management of this economically and ecologically important species.

## Figures and Tables

**Table 1:** Sequencing statistics using 454 sequencing platforms.

*Primary sequence data*			*No.*
Total number of reads			453,549
Total number of bases			221,052,902
Number of contigs			4,305
Number of bases			2,024,317
Number of Singleton			307,931
Total number of reads after trimmed			288,854
Total number of read-length after trimmed			148,321,261

***Di-nucleotide***	***No.***	***Di-nucleotide***	***No.***

AT	4,164	CA	6,544
CT	6,239	GC	86

***Tri-nucleotide***	***No.***	***Tri-nucleotide***	***No.***

AAA	8	TAC	8
AAT	273	TTG	124
AAG	164	TTC	102
AAC	62	TGA	65
ATA	238	TGG	124
ATG	53	TGC	104
ATC	89	TCG	3
AGA	85	TCC	134
AGT	10	GAG	117
AGG	98	GAC	6
AGC	82	GTG	54
ACA	56	GGG	10
ACG	2	GGC	32
ACC	90	GCG	26
TAA	261	CAG	103
TAG	14	CGG	28

**Table 2:** Characteristics of microsatellite loci from *Raja pulchra*.

Locus	Primer Sequence (5′→3′)		Motif	AT	Allele Size	No. of Allele	GenBank Accession No.
Rp03-nfrdi	F	**6-FAM** ACTGCCTAGGATGATGATGAAG	(AT)_13_	54	94–106	4	JQ433555
	R	TCTATATCCCTCCACTTCCTTG					
Rp11-nfrdi	F	**6-FAM** ATACACTCATCACTCACACCCC	(CA)_15_	61	108–134	10	JQ433556
	R	GTGGGTTAGTGCTCTTGTTCTC					
Rp16-nfrdi	F	**6-FAM** AGGAAGGCTTCAGCACATAAT	(TG)_13_	54	102–108	4	JQ433557
	R	CTCATCTGGAAGAGCACACAC					
Rp18-nfrdi	F	**6-FAM** ATTCCCTGATACAGATGGAGG	(CA)_16_	61	113–145	9	JQ433558
	R	TAAACTGTTTGCTCCTCTCTCC					
Rp19-nfrdi	F	**6-FAM** CAGACAATGAAACTCAACAGGA	(AG)_12_	54	96	1	JQ433569
	R	TCTAACTTCAATTAACCTTCGCA					
Rp22-nfrdi	F	**6-FAM** ATAGCATGAATACAATCCCAGG	(AG)_12_	54	102–108	3	JQ433559
	R	GATGATCACTTGGATTCCTGAT					
Rp24-nfrdi	F	**6-FAM** TGTTCTACAAGACACAAGGCAG	(AG)_12_	54	105–107	2	JQ433560
	R	ATTCCTCAGCTAACATCTCCAA					
Rp27-nfrdi	F	**NED** CATATTCATCATCAATTAAATCTGTC	(TG)_9_	54	224–232	3	JQ433561
	R	GCATATCCTTTGTCTGTCCAT					
Rp30-nfrdi	F	**NED** CGTGTATATGTATGTGTGCATGT	(TG)_11_	61	216–230	7	JQ433562
	R	GCAGAAGCACTACAGAATGTTT					
Rp34-nfrdi	F	**NED** TATGATCCATACAATCGCAAAA	(TG)_9_	54	240–250	6	JQ433563
	R	CAAATAGCAAACGACCTACACC					
Rp35-nfrdi	F	**NED** CTTACTGGTGAGGAATCTGAGC	(TG)_9_	61	226–236	5	JQ433564
	R	GCATACACTCCACACACCAC					
Rp39-nfrdi	F	**HEX** GCTTGGTTTTCTGAAATCAGTG	(AT)_13_	61	150–166	5	JQ433565
	R	ATAAAATTGCAGGGAGAATGC					
Rp43-nfrdi	F	**HEX** CTCCTGCCTTTGCTATGTGT	(TG)_15_	61	154–162	5	JQ433566
	R	GACTTTTCAGCGACAGTCTTCT					
Rp44-nfrdi	F	**HEX** ACATGGTCACGAGTAGAATGTG	(CA)_16_	54	149–161	6	JQ433567
	R	TTCAGACCCTATTCAAAATGCT					
Rp53-nfrdi	F	**HEX** GGACGGAATCCTTCTTTAAACT	(AG)_15_	54	140–148	5	JQ433568
	R	CTTTGTGCCTCTTTGTTAAACC					

**Table 3:** Variability at fourteen microsatellite loci in *Raja pulchra* populations from Korea.

Population		Microsatellite Loci														Mean of All Loci.
	
		Rp3 §	Rp11 §	Rp16	Rp18 §	Rp22	Rp24	Rp27	Rp30	Rp34	Rp35	Rp39	Rp43 §	Rp44	Rp53	
DC	*N*	28	30	30	30	30	30	30	30	30	30	30	29	30	30	29.8
	*Na*	3	7	4	7	3	2	3	6	6	5	4	5	6	5	4.7
	*A_R_*	3.00	6.64	3.83	6.78	3.00	2.00	2.83	5.80	6.00	5.00	4.00	4.97	5.64	5.00	4.61
	*R*	94–106	108–134	102–108	113–145	102–108	105–107	224–232	216–228	240–250	226–236	150–162	154–162	149–161	140–148	
	*Ho*	0.393	0.400	0.567	0.433	0.600	0.567	0.333	0.667	1.000	0.800	0.567	0.276	0.433	0.800	0.560
	*He*	0.511	0.690	0.584	0.676	0.671	0.503	0.288	0.666	0.788	0.773	0.657	0.477	0.443	0.769	0.607
	*F_IS_*	0.231	0.421	0.029	0.359	0.105	−0.126	−0.159	−0.002	−0.269	−0.035	0.138	0.422	0.022	−0.040	0.078
HS	*N*	29	30	30	30	30	30	30	30	30	30	30	25	30	30	29.6
	*Na*	4	10	4	8	3	2	2	6	6	5	5	4	5	5	4.9
	*A_R_*	4.00	9.79	3.98	7.62	3.00	2.00	2.00	5.81	6.00	5.00	5.00	4.00	4.80	4.83	4.84
	*R*	94–106	108–134	102–108	113–133	102–108	105–107	230–232	216–230	240–250	226–236	150–166	156–162	149–161	140–148	
	*Ho*	0.276	0.800	0.700	0.567	0.667	0.367	0.400	0.700	0.567	0.867	0.467	0.240	0.633	0.833	0.577
	*He*	0.603	0.797	0.595	0.732	0.608	0.481	0.364	0.676	0.746	0.801	0.494	0.487	0.573	0.702	0.619
	*F_IS_*	0.542 *	−0.004	−0.177	0.226	−0.097	0.237	−0.099	−0.035	0.241	−0.082	0.056	0.508 *	−0.104	−0.187	−0.002
Mean of All Pops.	*N*	28.5	30.0	30.0	30.0	30.0	30.0	30.0	30.0	30.0	30.0	30.0	27.0	30.0	30.0	
	*Na*	3.5	8.5	4.0	7.5	3.0	2.0	2.5	6.0	6.0	5.0	4.5	4.5	5.5	5.0	
	*A_R_*	3.50	8.22	3.90	7.20	3.00	2.00	2.42	5.81	6.00	5.00	4.50	4.48	5.22	4.92	
	*R*	94–106	108–134	102–108	113–145	102–108	105–107	224–232	216–230	240–250	226–236	150–166	154–162	149–161	140–148	
	*Ho*	0.334	0.600	0.633	0.500	0.633	0.467	0.367	0.683	0.783	0.833	0.517	0.258	0.533	0.817	
	*He*	0.557	0.744	0.589	0.704	0.639	0.492	0.326	0.671	0.767	0.787	0.576	0.482	0.508	0.736	
	*F_IS_*	0.387	0.208	−0.074	0.293	0.004	0.056	−0.129	−0.018	−0.014	−0.058	0.097	0.465	−0.041	−0.113	

Number of samples (*N*); number of alleles per locus (*Na*); allelic richness (*A_R_*); allelic size range (*R*); expected heterozygosity (*He*); and observed heterozygosity (*Ho*) are given for each population and locus.

* Not in conformity with Hardy-Weinberg equilibrium (*p* < 0.005, Bonferroni-corrected value).

§ Microsatellite loci revealed the presence of null alleles with MICRO-CHEKER 2.2.3.

**Table 4:** Screening of cross-species amplified microsatellite loci developed in *Raja pulchra* in related-species, skate and shark.

Species Name		N	Microsatellite DNA Markers										

			Rp3	Rp11	Rp16	Rp19	Rp24	Rp27	Rp34	Rp35	Rp39	Rp44	Rp53
**Rajiformes (Skate)**													
Rajidae	*Raja pulchra*	60	⦾	⦾	⦾	○	⦾	⦾	⦾	⦾	⦾	⦾	⦾
	*Okamejei boesemani*	3	⦾	⦾	⦾	○	⦾	⦾	X	⦾	⦾	⦾	⦾
	*Okamejei kenojei*	2	⦾	⦾	⦾	○	○	X	○	○	○	○	⦾
	*Okamejei acutispina*	3	○	⦾	⦾	○	○	X	○	⦾	⦾	⦾	⦾
Dasyatididae	*Dasyatis akajei*	1	X	○	X	○	X	X	X	X	X	X	○
	*Urolophus aurantiacus*	4	⦾	X	X	○	X	X	X	X	X	○	X
**Carcharhiniformes (Shark)**													
Scyliorhinidae	*Cephaloscylium isabaellum*	2	○	X	○	⦾	X	X	○	⦾	X	X	X
	*Scyliorhinus torazame*	1	○	○	○	X	X	X	X	○	X	X	X
Triakidae	*Mustelus manazo*	2	○	○	○	⦾	X	X	○	⦾	X	○	X
	*Triakis scyllium*	1	○	X	○	○	X	X	X	○	X	○	X

N: number of individual; ⦾: PCR amplified and polymorphic; ○: PCR amplified and monomorphic; X: non-PCR amplified.
